# Integrated Global Strategies for Toxoplasmosis Control Under the One Health Framework

**DOI:** 10.1155/tbed/3668308

**Published:** 2026-10-30

**Authors:** Yetian Li, Wuerguli Alimu, Kaiyu Xu, Weixin Gao, Zhendi Liu, Chenliang Jiang, Haoyi Yang, Xiaoyu Han, Zixun Yan, Pujie Jia, Bingrui Dai, Maolin Wen, Shuhan Zhang, Longhui Shen, Jishan Zheng, Jili Zhang

**Affiliations:** ^1^ Ningbo University School of Medicine Ningbo University Ningbo China nbu.edu.cn; ^2^ Ningbo University Affiliated Women and Children’s Hospital Ningbo University Ningbo China nbu.edu.cn

**Keywords:** environment, One Health, prevention, *Toxoplasma gondii*, zoonosis

## Abstract

*Toxoplasma gondii* is a globally distributed zoonotic parasite that infects approximately one‐third of the human population. Its intricate life cycle involving multiple hosts, combined with the remarkable environmental resilience of oocysts, renders it one of the most difficult pathogens to control. Toxoplasmosis imposes substantial public health and economic burdens, encompassing congenital infections, opportunistic diseases among immunocompromised individuals, reproductive losses in livestock, and persistent environmental contamination that perpetuates transmission. Conventional single‐sector interventions addressing human, animal, or environmental health in isolation have proven inadequate for sustainable control. The One Health framework offers an integrative approach that connects human, animal, and environmental health through cross‐sectoral collaboration, technological innovation, and community engagement. This review summarizes the epidemiological features and major transmission routes of *T. gondii*, and proposes comprehensive prevention and control strategies grounded in the One Health paradigm. Implementing this framework can bridge disciplinary boundaries, enhance early surveillance, and establish a robust, long‐term system to mitigate *T. gondii* transmission, thereby protecting human, animal, and ecosystem health.

## 1. Introduction

*Toxoplasma gondii* (*T. gondii*) is one of the most widespread zoonotic parasites globally, with an estimated global seroprevalence of 30%–50% in the human population [1, 2]. This obligate intracellular protozoan has the capacity to infect nearly all warm‐blooded animals [3, 4]. Its complex life cycle comprises three principal developmental stages, including tachyzoites, bradyzoites within tissue cysts, and oocysts, enabling the parasite to persist and propagate across various host species.

The disease burden of toxoplasmosis encompasses three interconnected domains, which are human health, animal productivity, and environmental sustainability. Infection is generally asymptomatic in immunocompetent individuals, but it can cause severe encephalitis, pneumonia, or chorioretinitis in immunocompromised individuals, such as those with AIDS or those undergoing organ transplantation [5–8]. In pregnant women, primary infection may result in miscarriage, stillbirth, or congenital toxoplasmosis, which can lead to an increased risk of long‐term neurological or ocular impairments in the offspring [9, 10].

From an agricultural and public health perspective, toxoplasmosis remains a globally distributed zoonosis with multifaceted impacts. Serological investigations have revealed high *T. gondii* infection rates in both livestock and companion animals, for instance, 9% in pigs and up to 75% in goats in Japan and 16% in domestic cats [11]. Such infections can lead to reproductive losses and measurable financial impacts; in Spain, *T. gondii*‐associated abortion outbreaks in sheep flocks caused direct economic losses of about €5000 per farm [12]. Environmental surveys further demonstrate the resilience of *T. gondii* oocysts, which have been detected in soil and water sources across Asia and Europe, indicating persistent contamination and cross‐species exposure risks [13–15]. These concurrent effects on animal health, economic productivity, and ecosystem integrity highlight the need for integrated prevention and control strategies within the One Health framework.

Historically, *T. gondii* control strategies have advanced in tandem with progress in knowledge and public health tools (Figure 1). In the mid‐20th century, early efforts prioritized prenatal screening and food safety, including thorough cooking of meat, to curb congenital infections [16]. As felids were later identified as definitive hosts, public health messaging turned to cat management and litter hygiene [17]. In the decades that followed, better diagnostics, research on livestock vaccination, and targeted public health education further lowered transmission risks in some contexts [18]. However, persistent issues like drug resistance, environmental oocyst resilience, and climate‐altered transmission patterns highlight that sustainable control requires an integrated One Health approach that links human, animal, and environmental interventions. Because *T. gondii* is characterized by multihost infection, diverse transmission routes, and environmental persistence, conventional sector‐specific prevention strategies are ineffective. Thus, implementing an integrated One Health framework is crucial. One Health is defined as a collaborative, multisectoral, and transdisciplinary approach that acknowledges the interconnectedness of humans, animals, plants, and their shared environment [17, 19]. Applying this framework to toxoplasmosis enables a comprehensive assessment of pathogen ecology and promotes coordinated control measures across medical, veterinary, and environmental sectors.

**Figure 1:**
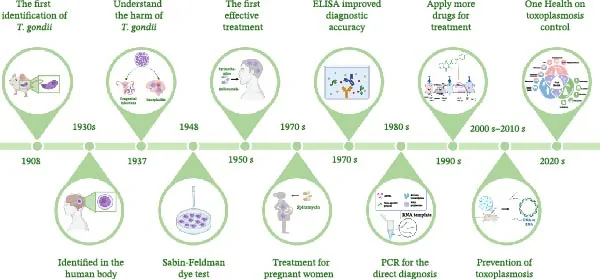
The history of toxoplasmosis control.

## 2. Epidemiology and Transmission Characteristics

### 2.1. Human Infection

Globally, the human seroprevalence of *T. gondii* shows substantial geographical heterogeneity. According to a recent large‐scale meta‐analysis covering 113 countries, Africa (42%) and South America (45%) have some of the highest population‐level seroprevalence estimates, while Australia also reports a notably high pooled rate of 54% [20]. Asia and North America generally report lower seroprevalence compared to other regions, though rates within Asia can vary widely by country [20–23]. Transmission primarily occurs through the ingestion of raw or undercooked meat containing tissue cysts, consumption of food or water contaminated with oocysts, or vertical transmission from infected mothers to their fetuses [22]. Less frequent transmission routes include organ transplantation and blood transfusion [24].

High‐risk populations include pregnant women, immunocompromised individuals, and occupational groups such as veterinarians, farmers, and butchers [5, 8, 9]. The disease burden is disproportionately concentrated in low‐ and middle‐income countries, where access to diagnostic services and antenatal care is limited. Congenital toxoplasmosis remains a significant public health challenge, especially in regions without routine prenatal screening [25–27]. A 2013 systematic review estimated that congenital toxoplasmosis causes ~190,100 cases worldwide each year, corresponding to a burden of 1.20 million disability‐adjusted life years (DALYs) annually [28]. In the absence of early detection, vertical transmission can lead to severe neurological and ocular damage in infants.

### 2.2. Animal Infection

*T. gondii* infects a wide range of domestic and wild animals, which act as reservoirs for zoonotic transmission. Felids, the sole definitive hosts, shed millions of oocysts following primary infection, thereby contaminating soil, water, and vegetation [14, 29]. While domestic cats typically exhibit mild or subclinical symptoms, wild and feral felids perpetuate transmission cycles in ecosystems largely outside human control.

In livestock, *T. gondii* infection leads to significant reproductive and economic losses. Pigs and small ruminants are particularly vulnerable, often experiencing abortion, stillbirth, or neonatal mortality [12]. The consumption of meat from infected animals remains a major source of human exposure. Additionally, wildlife species serve as intermediate hosts, sustaining *T. gondii* circulation within natural ecosystems and facilitating spillover to humans and domestic animals [14]. Marine sentinel species, such as sea otters, have provided further insights into these ecological dynamics, showing that oocysts transported by terrestrial runoff can persist in coastal environments and transmit across trophic levels [13].

### 2.3. Environmental Contamination

Environmental contamination is central to the persistence of *T. gondii* transmission. Oocysts can remain viable in moist soil for up to 18 months and in seawater for several months [15]. They are resistant to most standard disinfectants, including chlorine, and remain infectious under a variety of climatic conditions [15, 30]. Environmental contamination occurs through the feces of infected felids, carcasses of intermediate hosts, agricultural runoff, and sewage effluent [13, 31].

Climate change may further intensify this risk by altering rainfall patterns. In particular, intense rainfall events after dry periods can enhance the runoff‐mediated mobilization of oocysts accumulated in feces and soil, thereby increasing the risk of oocyst‐borne exposure in humans and animals [32].

### 2.4. Cross‐Sector Transmission Chain

The transmission of *T. gondii* forms a complex and interconnected “Human–Animal–Environment” network (Figure 2). Infected cats shed oocysts into the environment, and these oocysts are subsequently ingested by livestock during grazing, leading to the formation of tissue cysts that enter the human food chain. Meanwhile, human activities such as improper waste disposal or the consumption of undercooked meat may indirectly contribute to environmental contamination. These bidirectional feedback processes sustain transmission across hosts and ecosystems, maintaining endemic circulation.

**Figure 2:**
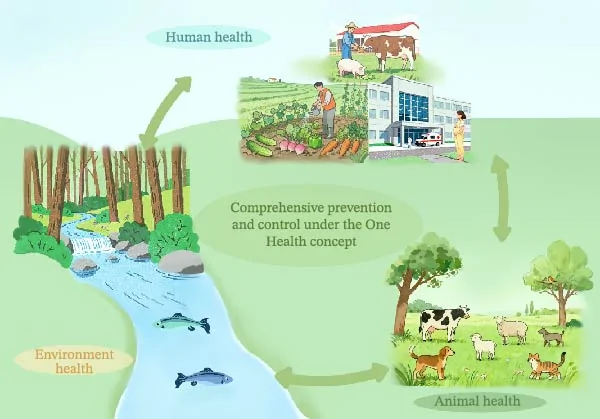
One Health components and their interactions for toxoplasmosis control.

Evidence suggests that control measures limited to a single sector, such as improving human hygiene, strengthening livestock management, or vaccinating cats, are often insufficient to achieve a substantial reduction in the overall infection risk. In contrast, integrated strategies that simultaneously address human, animal, and environmental pathways hold greater promise for interrupting the transmission and achieving sustainable control. This underscores the importance of cross‐sectoral collaboration and feedback‐driven governance, core elements of the One Health framework that provide the foundation for the comprehensive control strategies discussed in the following section.

## 3. Current Status and Limitations of Toxoplasmosis Control

Although substantial progress has been made in the diagnosis, treatment, surveillance, and risk prevention of toxoplasmosis across human, animal, and environmental dimensions, current intervention strategies remain fragmented and fail to achieve sustainable control of parasite transmission. A comprehensive evaluation of the limitations inherent in existing sector‐based approaches is therefore imperative to develop integrated One Health management frameworks.

### 3.1. Human‐Oriented Prevention and Limitations

Current human toxoplasmosis prevention is primarily tailored to high‐risk populations, with well‐established intervention protocols for pregnant women and immunocompromised individuals. For pregnant women, standardized prenatal screening is the core of clinical prevention. Serological tests at different gestational stages detect acute maternal infection for timely intervention. Early spiramycin use lowers mother‐to‐fetus transmission risk, and combined pyrimethamine‐sulfadiazine‐folinic acid treats confirmed fetal infection to optimize neonatal prognosis [25, 26, 33–35]. For immunodeficient patients, including HIV‐infected individuals and organ transplant recipients, regular serological monitoring, prophylactic medication, and targeted antitoxoplasma therapy effectively lower the risk of severe toxoplasmosis and mortality [36, 37]. In contrast, acute toxoplasmosis in immunocompetent individuals is typically self‐limiting and only requires symptomatic care, while public health prevention for the general population mainly depends on food safety education and behavioral risk avoidance [38, 39]. Diagnostic technologies, including ELISA and PCR, support early identification of infection across all populations, enabling timely clinical management [18, 40, 41].

Despite these targeted protective measures, human‐only prevention strategies have profound structural limitations. First, no licensed vaccine is available for human use, representing a critical gap in long‐term preventive strategies. Second, all approved therapeutic agents act exclusively against the tachyzoite stage of *T. gondii* and lack activity against latent bradyzoites in tissue cysts, meaning that current treatments cannot eliminate chronic infection or completely block transmission [36, 37]. Notably, chronic latent toxoplasmosis is closely associated with long‐term neuropsychiatric disorders and neurological sequelae, constituting a neglected public health burden [6]. Most existing strategies focus on acute infection in vulnerable groups while largely ignoring widespread chronic infection in the general immunocompetent population.

Based on the above context, the lack of preventive vaccines, the inability to eradicate latent parasites, and the insufficient coverage of chronic infection management severely restrict the capacity of human‐centered strategies to achieve sustainable population‐level control of toxoplasmosis.

### 3.2. Animal‐Oriented Prevention and Limitations

Animals occupy a critical position in the transmission cycle of *T. gondii*, involving both felids as definitive hosts responsible for oocyst shedding and farmed livestock as intermediate hosts, playing a key role in foodborne transmission. Therefore, current animal control measures mainly target these two groups.

For felids, interventions aim to reduce infection and oocyst shedding through host‐level management. For domestic cats, recommended measures include keeping cats indoors, feeding commercially prepared diets rather than raw meat, proper litter box management, and neutering [42, 43]. For free‐roaming and feral cats, population management, including spaying or neutering, has been proposed to reduce cat overpopulation and environmental contamination [42, 43].

In livestock populations, toxoplasmosis control relies primarily on farm biosecurity management to reduce exposure to oocysts. Major preventive strategies include safeguarding feed and water from contamination by cats and rodents, carrying out rodent control, enhancing sanitary conditions, and reducing outdoor exposure where feasible [12, 44]. Field intervention studies have demonstrated that such strategies can substantially reduce within‐farm *T. gondii* seroprevalence: in a crossover clinical trial on Dutch pig farms, cat neutering and feed coverage achieved estimated reductions of 67% (95% CI: 55%–78%) and 96% (95% CI: 77%–100%), respectively, within 1 year of implementation [44].

Although these animal‐based measures have been taken, the prevention and control of *T. gondii* still have significant limitations. Felid‐focused control measures, particularly those targeting free‐roaming or feral cats, are difficult to sustain at scale because population regulation and outdoor movement control cannot be carried out consistently [42, 43]. Biosecurity for livestock has limited generalizability across farming systems. Risk factors differ by species, housing conditions, outdoor access, feed and water management, rodent pressure, and the presence of cats around farms, making it difficult to apply a uniform control strategy across all livestock systems [12, 44]. In addition, information on the effectiveness and costs of farm‐level interventions remains limited. Although risk‐factor‐based measures have been proposed and some field interventions have shown encouraging results, more evidence is needed to evaluate which specific measures are effective, feasible, and cost‐effective under different farming conditions [44].

Collectively, the difficulty of sustaining felid‐focused control, the limited generalizability of livestock biosecurity, and the insufficient evidence on the effectiveness and costs of farm‐level interventions severely restrict the capacity of animal‐centered strategies to achieve sustainable transmission control of *T. gondii*.

### 3.3. Environmental Prevention and Limitations

Environmental contamination constitutes a critical interface in the transmission cycle of *T. gondii*. Oocysts shed by felids can persist in soil, be transported through water systems, and contaminate food products, thereby linking definitive hosts, environmental media, and human or animal exposure [13, 32]. Accordingly, current environmental prevention strategies can be broadly divided into source control and transmission pathway control.

Source control focuses on reducing the entry of oocysts into environmental media before widespread contamination occurs. Since felid feces are the direct source of environmental oocyst contamination, relevant measures include safe collection and disposal of cat feces, reducing fecal contamination in high‐risk areas such as gardens, sandboxes, children’s play areas, and farm surroundings, and managing local contamination hotspots [32, 42]. These measures aim to limit the deposition of oocysts into soil and other environmental sites where subsequent human, livestock, or wildlife exposure may occur.

Transmission pathway control focuses on limiting the movement of oocysts through water and food systems after environmental contamination has occurred. In water environments, preventive measures include protecting water sources from fecal contamination, reducing runoff‐mediated transport, and applying physical removal or advanced treatment processes where appropriate [13, 32]. Sand filtration and membrane filtration can contribute to oocyst removal in drinking water systems, while ultraviolet irradiation and ozone treatment have been investigated as additional inactivation methods [30, 32]. In food systems, prevention includes using safe irrigation and washing water, thoroughly washing and peeling fresh produce, and applying safe food‐handling practices before consumption [32]. Heat treatment, irradiation, and high‐pressure processing have also been evaluated as physical methods for inactivating *T. gondii* oocysts in food products [30].

Despite these environmental control measures, substantial limitations remain. First, environmental transmission is cross‐media in nature. Oocysts deposited in soil can be transported by rainfall and runoff into surface waters and may further contaminate irrigation water, fresh produce, seafood, and other food products, making contamination difficult to localize and control through isolated soil, water, or food interventions [13, 32]. Second, environmental management is difficult because contamination sources are widely distributed and often intermittent. Felid fecal deposition may occur in residential areas, farms, watersheds, and other outdoor environments, making it difficult to identify and manage all contamination sites continuously [32, 42]. Third, technical limitations continue to weaken environmental surveillance and control. Oocysts in soil, water, fresh produce, and seafood are often unevenly distributed and may occur at low concentrations, making detection and quantification difficult [32]. Moreover, their resistance to many chemical treatments limits the effectiveness of conventional disinfection, while the performance of physical or advanced treatment methods still depends on environmental matrices, oocyst load, and treatment conditions [30, 32].

Overall, the broad cross‐media dispersal of oocysts, the difficulty of continuously managing diffuse environmental contamination sources, and the technical limitations in detecting and inactivating oocysts severely restrict the capacity of environment‐centered strategies to achieve sustainable control of *T. gondii* transmission.

Taken together, a critical review of existing prevention and control measures reveals persistent gaps in coordination, scientific innovation, and management effectiveness across the human, animal, and environmental domains. After comparing all measures and effects on humans, animals, and the environment, human‐targeted measures, such as prenatal screening, hygiene education, and safe food handling, contribute most to reducing *T. gondii* infection rates because they directly interrupt the final transmission pathway to people, preventing severe outcomes like congenital toxoplasmosis. However, the persistently high prevalence suggests that human‐focused actions alone are insufficient. Greater efforts are needed to control the infection rate of *T. gondii* in animals and the environment. This highlights the value of a more integrated One Health‐based approach to better address the transmission across these interconnected interfaces.

## 4. Integrated Control Strategies Based on the “One Health” Concept

### 4.1. “One Health” Concept and Its Advantages

The One Health framework integrates human, animal, and environmental health through coordinated surveillance and intervention, which is essential given the complex multihost transmission of *T. gondii*. Compared with fragmented strategies, it enables cross‐sectoral data sharing and joint risk assessment, improving timeliness, coherence, and efficiency in identifying and controlling transmission, whereas conventional siloed systems often delay detection and response.

In recent years, One Health has seen significant progress through advancements in research methodologies, technology, and interdisciplinary collaboration to address complex health challenges. Predictive tools and modeling techniques have improved alongside innovations in surveillance and control strategies, allowing us to better anticipate, detect, and mitigate the spread of zoonotic diseases, such as vector‐borne illnesses and emerging infectious diseases like COVID‐19 [45]. For example, advances in predictive analytics and machine learning algorithms enable the integration of diverse datasets from human, animal, and environmental sources [19].

### 4.2. Integrated Control Strategies for Toxoplasmosis

The concept of One Health has been developed as the appreciation that human health is intricately connected to that of other animals and the environment that they inhabit. The United Nations Sustainable Development Goals are relevant to One Health as they include targets for health and well‐being, clean water and sanitation, climate action, as well as sustainability in marine and terrestrial ecosystems [46, 47]. Therefore, scientists all over the world attach great importance to the development of One Health in zoonotic diseases, and certain achievements have been made in some countries and certain fields. We have roughly classified these measures into four categories: multisectoral collaboration mechanism, public participation and health education, multidisciplinary technological innovation support, and policy support or governance system integration.

Multisectoral collaboration can integrate measures at various management levels to address intractable challenges related to *T. gondii* transmission in the environment. Public health education can raise public awareness of toxoplasmosis and enhance the proficiency of professionals, thereby mitigating the limitations in current human and animal toxoplasmosis control. Multidisciplinary technological innovation supports the development of novel anti‐*T. gondii* drugs and vaccines for the progressive management of chronic infections. Moreover, it can also be integrated with other disciplines to make the detection and prevention of *T. gondii* more accurate. Finally, policy support and governance‐system integration enable human, animal, and environmental health to be incorporated within a unified strategy, resolving problems that cannot be addressed by single‐sector approaches (Figure 3).

**Figure 3:**
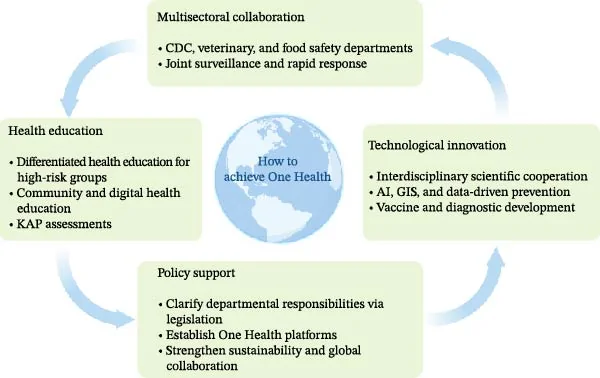
Key strategies for achieving One Health in toxoplasmosis prevention.

#### 4.2.1. Multisectoral Collaboration Mechanism

Guided by the One Health principle that prioritizes interdisciplinary collaboration and public participation [48], a unified framework for *T. gondii*’s control is essential, in which different sectors coordinate their strengths to achieve integrated disease prevention goals.

To operationalize such structured cross‐departmental collaboration in practical zoonotic disease management, it is critical to establish a clear institutional division of labor and regularized interagency linkage mechanisms, as exemplified by Ethiopia’s national multisectoral One Health surveillance and response system. This generalized zoonotic disease governance framework unifies the responsibilities of three core governmental institutions: the Ministry of Health (MOH), the Ministry of Agriculture (MOA), and the Ethiopian Wildlife Conservation Authority (EWCA), covering cross‐host disease transmission involving humans, livestock, and wildlife. Within this integrated framework, the Ethiopian Public Health Institute (EPHI) under the MOH oversees human disease surveillance, epidemic investigation, and laboratory pathogen detection; MOA’s professional departments cooperate with the National Animal Health Diagnostic and Investigation Center (NAHDIC) to implement routine monitoring and risk control for livestock‐borne diseases; and EWCA takes charge of wildlife habitat protection and wild animal health management to mitigate wildlife‐associated disease risks [17, 49, 50]. Although this established model targets general zoonotic diseases rather than *T. gondii* specifically, its standardized cross‐sectoral collaboration paradigm illustrates the core operational logic of the One Health approach. It provides a valuable referential template that can be adapted and optimized for targeted *T. gondii* prevention and control worldwide.

Drawing on the universal cross‐sectoral collaboration logic of One Health demonstrated in Ethiopia’s generalized zoonotic disease governance framework, policymakers can establish targeted multidepartmental linkage mechanisms for specialized *T. gondii* prevention and control. Adapted to the multihost transmission features of toxoplasmosis, a refined collaborative system involving public health authorities, food safety supervision departments, and animal veterinary institutions enables full‐chain management of *T. gondii* transmission. Specifically, public health agencies deploy digital monitoring systems to integrate population, animal, and environmental data for epidemic prediction and targeted intervention [51]. Food safety departments prioritize routine *T. gondii* detection in high‐risk animal‐derived food products, while customs enforce strict border inspections to curb cross‐regional pathogen transmission. Meanwhile, veterinary authorities have built standardized livestock epidemic prevention systems to reduce animal infection rates at the source [51]. The coordination of these multilevel, toxoplasmosis‐specific measures achieves systematic whole‐process supervision, markedly enhancing the efficiency of One Health‐based integrated prevention and control.

#### 4.2.2. Public Participation and Health Education

Effective *T. gondii*’s prevention relies not only on governmental supervision but also on extensive public participation. As human, animal, and environmental health are intrinsically interconnected under the One Health framework, improving public health awareness and engagement is essential for sustainable toxoplasmosis control.

Sustainable grassroots toxoplasmosis governance depends on both professional capacity building and targeted public health education, as reflected in Ethiopia’s mature One Health practices. To strengthen local zoonotic disease response capacity, Ethiopia has established a systematic talent training system centered on the Center for Disease Control and Prevention (CDC)‐based Field Epidemiology Training Program (FETP) and long‐term university collaboration platforms. By integrating One Health principles and infectious disease research into professional training, this model effectively improves grassroots practitioners’ competencies in cross‐domain epidemic governance [17, 52]. The strengthened professional workforce supports precise *T. gondii* intervention, while hierarchical, differentiated health education targeting high‐risk groups, students, and clinical and veterinary personnel expands social prevention coverage. Drawing support from multichannel media promotion and regular knowledge, attitude, and practice (KAP) evaluations to optimize educational strategies [50], the combination of professional empowerment and public engagement facilitates the construction of a sustainable social governance system for toxoplasmosis control.

Referencing the above practical experience, authorities should implement targeted, tiered health education and public mobilization for *T. gondii* prevention and control. In view of the different infection risks of pregnant women, pet breeders, livestock workers, and other high‐risk groups, differentiated health publicity and education schemes should be formulated. Meanwhile, the popularization of the One Health concept should be normalized and universalized: integrating One Health and toxoplasmosis‐prevention knowledge into school curricula, training for physicians and nurses, and veterinary professional teaching, and using television, new‐media platforms, and other diverse communication channels to deliver full‐coverage health education across urban and rural regions [53]. KAP assessments can dynamically optimize educational content and publicity methods, effectively improve the public’s awareness of the correlation between human, animal, and environmental health, and guide the public to form healthy living and breeding habits. This social governance model combining professional guidance and public participation can effectively reduce the human‐induced *T. gondii* transmission risks and support the long‐term sustainable prevention and control of toxoplasmosis.

#### 4.2.3. Multidisciplinary Technological Innovation Support

Prevention and control of *T. gondii* require clinical and public health efforts and multidisciplinary collaboration. Under the One Health framework, professionals from medical, veterinary, environmental, pharmaceutical, and data science fields synergize surveillance, research, and interventions to enhance epidemic analysis, drive innovation, and strengthen overall management capacity.

With the rapid development of digital technology, the Geographic Information System (GIS) has become a powerful tool for toxoplasmosis monitoring. By integrating spatial data analysis with diagnostic techniques, GIS facilitates the assessment and prediction of regional *T. gondii* prevalence. For instance, Vitomir adopted GIS‐based predictive modeling and identified western Serbia as a high‐risk area for ovine toxoplasmosis, providing reliable spatial evidence for local epidemic prevention despite insufficient field data [54, 55]. Consistently, Francisco proposed that effective *T. gondii* monitoring requires the integration of laboratory detection, molecular and epidemiological techniques, environmental monitoring, and cross‐institutional data sharing during international academic symposia [56]. Such multitechnology synergy enables a visualized and refined assessment of regional epidemic risks, helps compensate for the limitations of single‐dimensional monitoring strategies, and provides robust technical support for precise One Health intervention.

Technological innovation and real‐time risk surveillance are essential to translate theoretical research into practical public health strategies for the prevention and control of toxoplasmosis. GIS enables spatial analysis of infection patterns, demographics, and environmental determinants for hotspot identification and outbreak prediction [57, 58]. Artificial intelligence (AI) enhances these systems via automated data integration, early warnings, and decision‐making. In addition, advances in vaccines, diagnostics, and therapeutics are key to this multidisciplinary approach. The biopharmaceutical industry should accelerate the R&D of molecular diagnostics, vaccines, and targeted therapies with optimized formulations to reduce environmental residues. Notably, experimental live‐attenuated feline vaccine candidates, such as the *T. gondii* HAP2 knockout strain, have been shown to reduce oocyst shedding upon wild‐type challenge in preclinical studies [59]. Although commercial feline vaccines remain unavailable, emerging mRNA and multivalent human vaccine platforms have yielded encouraging preclinical results, and cross‐disciplinary cooperation between medical and veterinary disciplines is crucial for promoting the clinical translation of these innovative findings [60].

#### 4.2.4. Policy Support and Governance System Integration

Effective control of *T. gondii* requires sound One Health policies to integrate human, animal, and environmental health governance. Governments should clarify departmental responsibilities for surveillance, risk assessment, emergency response, and health education via legislation. Policy incentives can promote cross‐sector collaboration in research, healthcare, veterinary, and public health fields, while standardized data sharing ensures consistent, evidence‐based interventions [61, 62].

Global organizations have strengthened unified One Health governance. The World Health Organization (WHO), the World Organization for Animal Health (WOAH—formerly OIE), the UN Food and Agriculture Organization (FAO), and the UN Environment Programme (UNEP) jointly constitute the One Health Quadripartite to promote global sustainable development through cross‐domain cooperation [19]. The One Health model has been maturely applied to antimicrobial resistance control, with targeted regulations on food management, public health intervention, and epidemic surveillance to reduce health hazards [63]. Likewise, countries can adopt this integrated multistakeholder framework to formulate systematic policies for toxoplasmosis prevention.

Sound institutional mechanisms ensure the sustainable control of *T. gondii*. Incorporating One Health into national public health governance and establishing standardized monitoring, evaluation, and accountability systems can sustain adaptive and effective disease management [61, 64]. Additionally, engagement of the private sector and international organizations should be encouraged to diversify funding sources and enhance the sustainability and global collaboration of *T. gondii* control programs [64, 65].

Overall, efficient management of *T. gondii* demands a transition from fragmented single‐department intervention to integrated One Health governance. This holistic model unifies surveillance technologies and policy strategies based on the interconnection of human, animal, and environmental health. Cross‐sector cooperation bridges data gaps, standardizes diagnosis and prevention protocols, and improves regional and national coordination. The integrated One Health system accelerates the translation of research findings into practice and enhances the global early warning and sustainable control capacity for toxoplasmosis.

## 5. Conclusion

*T. gondii* remains a persistent zoonotic challenge due to its complex transmission dynamics and environmental resilience. Traditional single‐sector control strategies are inadequate for sustainable management. Integrating human, animal, and environmental health within the One Health framework offers a more effective pathway forward. Future efforts should prioritize the development of cross‐species vaccines, the enhancement of molecular diagnostics, and the establishment of data‐sharing platforms to strengthen surveillance and risk prediction. Policy coordination, public education, and intersectoral collaboration are vital to translating these advances into practice. Through science‐driven governance and sustained cooperation, One Health‐based control can significantly reduce the global burden of toxoplasmosis and protect both human and ecosystem health.

## Funding

This research was funded by the Ningbo Municipal Bureau of Science and Technology Project (Grant 2025Z153), the Ningbo Natural Science Foundation (Grant 2024J465), the Double First‐Class Cooperation Project of Ningbo University (Grant HX2026000110), the Li Dak Sum, Yip Yiu Jen, & Li Pun Jun Marine Biopharmaceutical New Drug R&D Special Project (Grant 2026‐01), the High‐Level Scientific Research Project of Ningbo University Project (Grant GJPY2026042), the Ningbo Leading Medical & Health Discipline (Grant 2026‐A34), and the Ningbo Clinical Research Center for Children’s Health and Diseases (Grant 2019A21002).

## Conflicts of Interest

The authors declare no conflicts of interest.

## Data Availability

The authors have nothing to report.
